# Per- and polyfluoroalkyl substances (PFAS) and heavy metals in the egg of peregrine falcon (*Falco peregrinus*) populations in West England, United Kingdom

**DOI:** 10.1007/s10646-026-03076-x

**Published:** 2026-04-11

**Authors:** Shinji Ozaki, Jacqueline S. Chaplow, Beverley Dodd, Helen Grant, M. Glória Pereira, Elaine Potter, Richard G. Sale, Darren Sleep, Sarah Thacker, Steve J. Watson, Lee A. Walker, Suzane M. Qassim

**Affiliations:** 1 UK Centre for Ecology & Hydrology, Library Avenue, Bailrigg, Lancaster, LA1 4AP UK; 2 Independent researcher, Coberley, GL53 9QY UK; 3 South-West Peregrine Group, Old Builders Arms, Randalls Green, Chalford Hill, Stroud, Gloucestershire, GL6 8EF UK; 4 Natural England, 4th Floor, Eastleigh House, Upper Market Street, Eastleigh, SO50 9YN UK

**Keywords:** Apex predator, Eggshell index, long-chain Perfluoroalkyl acids, PBT substances, Stable isotope

## Abstract

This study focused on the exposure of a terrestrial raptor, the peregrine falcon (*Falco peregrinus*), in the United Kingdom. In contrast to inland areas, peregrine falcons in coastal areas of North Cornwall, South-west England, have recently declined despite a decreasing trend in environmental legacy organic contaminants. Exposure to per- and polyfluoroalkyl substances (PFAS) is suspected to contribute to one of the causes of this decline. However, unlike studies on aquatic birds, research on PFAS exposure of terrestrial predatory birds remains limited, particularly in British wild birds. To fill this knowledge gap, we have measured PFAS burdens in peregrine eggs from different English areas and compared them with stable isotope and eggshell index values.

Our results showed that long-chain perfluoroalkyl acids were predominantly detected in peregrine eggs. Perfluorooctane sulfonyl acid (PFOS), perfluorohexanesulfonic acid (PFHxS), and perfluorooctanoic acid (PFOA) residues significantly differed among counties: PFOS and PFHxS residues were significantly higher in eggs from Devon, the study area around urban settlements, than in Cornwall. PFOA residues were significantly higher in Lancashire, an inland study area, than in Devon. Several perfluoroalkyl carboxylic acid residues showed significant and negative correlations with δ^13^C in eggs, suggesting that the sources of these PFAS might come from terrestrial habitats. No significant relationship was observed between eggshell index and PFAS residues. Given the variation in PFAS exposure among areas, it remains challenging to determine the impact of PFAS on the Cornwall peregrine population. Further studies are needed to fill these knowledge gaps.

**Supplementary Information:**

The online version contains supplementary material available at 10.1007/s10646-026-03076-x.

## Introduction

Per- and polyfluoroalkyl substances (PFAS) are a class of many thousands of substances (Buck et al. 2011; OECD 2018) applied for over 70 years to a broad range of industrial usage like fluoropolymer manufacture and to various commercial products such as water repellents, food-contact materials, and aqueous film–forming foams (AFFFs) (Glüge et al. 2020; Kissa 2001). Numerous studies have detected their ubiquitous distribution in the environment (Evich et al. 2022) and biota (Ahrens and Bundschuh 2014; Giesy and Kannan 2001; Houde et al. 2006), as well as their toxicity for humans (Fenton et al. 2021). In the late 1990s and early 2000s, many studies focused on long carbon-chain (LC-) perfluoroalkyl acids (PFAAs), such as perfluorooctane sulfonyl acid (PFOS) and perfluorooctanoic acid (PFOA), to clarify their toxicological and ecotoxicological aspects. As a result, these substances have been identified as persistent, bioaccumulative, and toxic (PBT) or very persistent and very bioaccumulative (vPvB) substances (OECD 2013). Regulatory measures, industry voluntary initiatives, and risk management programmes restricting or eliminating releases of LC-PFAAs and their precursors have been initiated since then, particularly in many developed countries, to mitigate their adverse effects on humans and the environment. For example, 3M Company, a main producer of PFAS, phased out the production of PFAAs with eight carbons (C8-PFAAs; i.e., PFOA, PFOS, and their related products) during 2000 − 2002 (3M Company 2000). Moreover, PFOS and its related products have been regulated in European Union (EU) since 2006 (European Parliament and The Council, 2006) and were added to Annex B of the Stockholm Convention on Persistent Organic Pollutants (POPs) in 2009 that restricts their use (UNEP 2009). PFOA and perfluorohexane sulfonic acid (PFHxS) were also listed in the Stockholm Convention later (UNEP, 2019, 2009). Although production of these PFAS has decreased in Europe since then (Paul et al. 2009), PFOS and other PFAS regulated in Europe are still produced in some countries, such as China (Jia et al. 2023; Zhang et al. 2012). Meanwhile, industrial transitions have replaced these LC-PFAAs and their precursors with alternative chemicals, but PFAAs are released from the degradation of these alternative chemicals (Buck et al. 2011; Wang et al. 2017). Therefore, exposure to PFAS and their undesired adverse effects remains a serious issue.

Birds of prey have been widely considered as sentinels for biomonitoring of environmental pollution because of their longevity, wide range of territory, and high trophic levels within food webs (Furness 1993). PFAS exposure in wildlife has been largely reported with aquatic predatory birds, such as guillemots (Holmström and Berger 2008; Löfstrand et al. 2008), cormorants (Herzke et al. 2009; Kannan et al. 2002; Nordén et al. 2013), gulls (Blévin et al. 2017; Gebbink and Letcher 2010), and gannets (Pereira et al. 2021), while studies on terrestrial predatory birds have recently arisen but remain limited (Ahrens et al. 2011a; Bustnes et al. 2015, 2022; Eriksson et al. 2016; Groffen et al. 2023; Holmström et al. 2010; Jaspers et al. 2013). In general, differences in PFAS exposure between terrestrial and aquatic birds could result from various factors related to their movement and transfer, such as chemical properties, releases from primary sources, exposure pathways, and the trophic positions of prey and predators. For instance, PFAS can be released to the aquatic environment through leachate from landfills and sewage sludge-amended soils, effluent from industry and domestic wastewater, and runoff from PFAS-based firefighting, while they are also emitted into the air and deposited on soil for the terrestrial environment (Health and Safety Executive, 2023). Short-carbon chain (SC-) ionic PFAS show high water solubility and are likely to end up in the aquatic environment (Health and Safety Executive, 2023; Mahinroosta and Senevirathna 2020), while atmospheric transport can also contribute to the dispersal of PFAAs, notably precursors of PFAAs like fluorotelomer alcohols (FTOHs) (Bustnes et al. 2015; Prevedouros et al. 2006). LC-PFAAs are considered bioaccumulative, whereas SC-PFAAs are not (Buck et al. 2011; Fremlin et al. 2023; OECD 2013), which results in high levels of PFAS burdens, particularly for PFAAs with a carbon chain longer than eight carbons (>8C PFAAs), in top predators (Androulakakis et al. 2022; Conder et al. 2008; Gebbink and Letcher 2012). Given that these factors vary between terrestrial and aquatic ecosystems, the PFAS exposure pattern of water predatory birds is not, at least not directly, extended to terrestrial birds. There are still important knowledge gaps in PFAS exposure of terrestrial animals.

The peregrine falcon (*Falco peregrinus*) is a raptor observed in a wide range of terrestrial habitats in the Northern Hemisphere, such as mountain areas, cliffs, river valleys, coastlines, and also urban or semi-urban areas (Sale 2016). This predatory species hunts a diverse range of prey from both aquatic and terrestrial habitats, depending on the prey availability. In the United Kingdom (UK), although pigeons are their preferred food item nationwide, peregrines in coastal areas also prey on several seabirds, like gulls (*Larus fuscus*, *Larus argentatus*, *Chroicocephalus ridibundus*) and razorbill (*Alca torda*) (Dixon et al. 2018; Sale 2016; Sutton 2015). It is also one of the iconic species for terrestrial biomonitoring because, like other raptor populations, peregrine falcons have been shown to be sensitive to the adverse effects of chemical contaminants, such as organochlorine insecticides (Castagna et al. 2024). In the UK like other countries, this species was critically endangered in the mid-1940s due to high concentrations of organochlorine insecticides that biomagnify through the trophic chain, affecting the eggshell thickness and thus the reproduction success (Mearns and Newton 1988; Newton et al. 1989). After the prohibition of certain highly toxic pesticides in the late 1970s, such as DDT, peregrine falcon populations have recovered in the UK (Oli et al. 2023; Ratcliffe 1993). However, the study of Watson and Sale (2020) recently demonstrated a reduced breeding success in peregrine populations in coastal North-west Cornwall, South-west (SW) England: the numbers of fledglings per clutch and per monitored site have been gradually declining since the 2010s. As no comparable trend was observed in inland areas, these authors suggested that marine pollution was a potential candidate for the decline in coastal populations. Meanwhile, other recent studies have reported a decrease in legacy persistent organic contaminants, such as PCBs and DDT, in aquatic birds in England, Wales, and/or Ireland (Kean et al. 2021; Power et al. 2021a, b). The decline in the Cornwall peregrine populations may be due to exposure to other contaminants, such as PFAS, given the potential impacts of high PFAS exposure on the health of both terrestrial and aquatic birds (Groffen et al. 2019; Lau et al. 2004; Letcher et al. 2010; Lopez-Antia et al. 2019; Nøst et al. 2012). However, studies focusing on PFAS in the UK wildlife are currently few, such as northern gannets (*Morus bassanus*) (Pereira et al. 2021) or Eurasian otters (*Lutra lutra*) (O’Rourke et al. 2022, 2024), and only limited data on PFAS exposure of UK terrestrial predatory birds are available (Health and Safety Executive, 2023).

Given the knowledge gap on PFAS in UK terrestrial birds, one aim of this study is to report PFAS exposure levels in UK peregrines from different regions using their eggs. Heavy metals in eggs were also measured to assess their potential impact on the population decline following a recent report of high mercury (Hg) concentrations in the eggs of guillemots (*Uria aalge*) from Ireland (Power et al. 2021b). Stable isotopes, which are widely used in animal foraging and spatial distribution studies, were measured in eggs and compared with contaminant residues as parameters characterising peregrines’ diet and habitat characteristics. Different dietary items often have different isotope signatures, which is reflected in the tissues of consumers (Wiley et al. 2017). The stable ratios of carbon, that reflect different carbon pools of producers (e.g., terrestrial C3, C4, and CAM plants or aquatic algae), and of nitrogen, that is an effective tracer of trophic level, are widely used in avian ecology (Inger and Bearhop 2008). We also aim to assess possible eggshell thinning in peregrines in response to environmentally relevant concentrations of PFAS and metals. Several studies have observed adverse effects of PFAS exposure on reproduction in birds (Custer et al. 2014; Molina et al. 2006), including eggshell thinness in great tits (*Parus major*) (Groffen et al. 2019). There are different mechanisms of chemically induced eggshell thinning (de Solla et al. 2023). For example, eggshell thinning caused by DDE or certain NSAIDs, such as diclofenac, is mainly due to inhibition of cyclooxygenase (COX) synthesis, enzymes responsible for forming lipid-based signalling molecules involved in calcium excretion (de Solla et al. 2023). The inhibition of carbonic anhydrase (CA) that catalyses carbonate ion formation also causes eggshell thinning by exposure to oestrogens (Berg et al. 2004; Holm et al. 2006). Following the recent report of human carbonic anhydrase activity inhibition by PFAS (Nguyen et al. 2020), we assessed the relationship between eggshell thickness and chemical residue concentration as a possible impact on bird reproduction.

## Materials and methods

### Peregrine egg collection and preparation for chemical analysis

Failed and deserted peregrine eggs were collected in three sites in England: Cornwall, Lancashire, and Devon and Dorset, under research by the British Trust for Ornithology (BTO) (https://www.bto.org/). The nests in Cornwall were located near coastal cliff areas in SW England, whereas the nests in Lancashire, North-west England, were in an inland area, which reflects a contraction between peregrine populations from coastal and inland areas (for the location of the sampling points, see Supplementary Information SI Fig. 1). The nests in Devon and Dorset, referred to as ‘Devon’ hereinafter, were located near urban areas of Exeter and Bournemouth, respectively, that are close to the English Channel, an arm of the Atlantic Ocean separating Southern England from northern France. The hunting range of the UK peregrine from their eyrie in the breeding season is limited (Ratcliffe 1993; Sale 2016), and UK female peregrines showed 22–117 km^2^ of hunting range in the study of Mearns (1985). Although there is no precise data on the diets of peregrines across the three sites, we assumed that the two sites in South England (i.e., Cornwall and Devon) would provide different food resources.

Eggs were procured under the BTO Schedule 1 Licence. They were obtained by licensed workers’ hand direct from the respective eyries as a by-product of ringing peregrine chicks, using climbing ropes where necessary. Egg samples were then submitted to the Predatory Bird Monitoring Scheme (PBMS; https://pbms.ceh.ac.uk/) and stored at 4 °C by the PBMS before extracting egg content. In this study, we used 20 failed and deserted eggs collected between 2014 and 2018 from 15 nests from the three sites (five nests per site). Some eggs were collected from the same nests: four eggs were collected from one of the nests in Cornwall, two eggs were from one of the nests in Devon, and two other eggs were from another nest in Devon. This pseudo-replication issue was dealt with in our data analysis.

When we sampled egg contents from each egg, a macroscopic inspection of the egg content was performed to check the embryo development, and no visible abnormal embryo development was observed in our egg samples. Egg content was then collected and homogenised using a blender (Laboratory mixer emulsifier, Silverson Machines Ltd.) and stored in glass jars with polypropylene caps at -20 °C until chemical analyses. The moisture content of the sample was determined by drying a 1 g sub-sample at 70 °C for at least 24 h. The average moisture content of the 20 peregrine egg samples was 80.8% (range: 78.1–85.3%). Based on the moisture content data, we assumed that these eggs were not desiccated.

### Eggshell index

The eggshell thickness was assessed according to Cooke (1973) to calculate the eggshell index. The measurement tolerance was 0.1 mm. The eggshell was rinsed, left to dry for four weeks at ambient temperature, then weighed. An eggshell thickness index (ESI) was calculated for each egg according to Ratcliffe (1970): ESI = shell weight (mg) / (length (mm) x breadth (mm).

### Analysis of PFAS in peregrine eggs

In this study, we measured 13 perfluoroalkyl carboxylic acids (PFCAs: PFBA, PFPeA, PFHxA, PFHpA, PFOA, PFNA, PFDA, PFUdA, PFDoA, PFTrDA, PFTeDA, PFHxDA, PFODA), three perfluorosulfonic acids (PFSAs: PFBS, PFHxS, PFOS) and perfluorooctane sulfonamide (PFOSA) in peregrine eggs. (For the information on each PFAS, see SI Table 1).

Approximatively 1 g of a single wet sample of homogenised whole egg content was spiked with ^13^C labelled standards (PFBA, PFHxA, PFHxS, PFOA, MPFNA, MPFOS, MPFDA, MPFUdA, MPFDOA, MPFTeDA; Wellington Laboratories, Ontario, Canada), and 1 mL of acetonitrile was added. The samples were incubated for 19 h at 4 °C. After incubation, another 8 mL of acetonitrile was added, and the samples were thoroughly mixed using a vortex for 10 min three times. Samples were then centrifuged at 2000 rpm for 5 min, and the supernatant was collected and evaporated to dryness. The residue was resuspended in acetonitrile, re-dissolved in acetonitrile and vortexed. The extracts were cleaned by adding 50 mg of activated carbon, which were vigorously mixed for 1 min. Samples were centrifuged for a further 15 min at 5000 rpm, and then the supernatant was removed and evaporated to dryness. Samples were reconstituted in the mobile phase used for liquid chromatography-mass spectrometry (LC-MS/MS). PFAS concentrations in eggs were analysed with liquid chromatography coupled to a triple quadrupole mass spectrometer (Waters ACQUITY UPLC system coupled with a Waters XEVO TQ-XS Tandem Mass Spectrometer; UK), interfaced with a Unispray source in negative multiple reaction monitoring (MRM) mode and operated using Masslynx software (version 4.2). 5 µL were injected using an Acquity UPLC BEH C18 analytical column (1.7 μm particle size, 100 mm x 2.1 mm, Waters, USA). The mobile phase consisted of (A) LCMS grade water with 10 mM ammonium acetate (80:20) and (B) methanol with 10 mM ammonium acetate. Gradient elution started from 80%A and 20%B, increased to 45%B in 6 min and to 95%B in 14 min and held for 3 min, at a flow rate of 0.3 mL min^-1^. For quality control and assurance purposes, a blank and a spiked chicken egg were included in each batch. No PFAS was detected in the blank. The performance of the method was assessed in terms of the limit of quantification (LoQ), recovery of the internal standards for the analytes and linearity. Recovery for the total procedure was calculated using the labelled standards.

Concentrations of PFAS in eggs are expressed as ng g^-1^ of wet weight (ww). The average recoveries ranged between 78 and 94% (SI Table 1). The limit of quantification (LoQ) was estimated using the lowest detectable calibration standard concentration. The mean LoQ for the PFAS was 0.3 ng g^-1^ ww and ranged from 0.02 to 0.08 ng g^-1^ ww (SI Table 1).

### Analysis of metals in peregrine eggsAnalysis of metals in pereg

We also measured concentrations in eggs of Hg, cadmium (Cd), lead (Pb) as other legacy contaminants. Selenium (Se) was also analysed because of its antagonistic interaction with Hg, and a Hg/Se molar ratio approaching 1 is considered protective of Hg toxicity due to the existence of mercuric selenide (HgSe) (Cuvin-Aralar and Furness 1991; Sumino et al. 1977; Yang et al. 2008). Other elements in peregrine eggs were also measured in the same analytical process: arsenic, nickel, chromium, copper, zinc, iron, manganese, cobalt, strontium, and molybdenum. The results of these elements are shown in SI Table 2.

Approximatively 1 g wet weight sample of egg content was digested in 10 mL of 70% ultrapure nitric acid (Baker, Ultrex II) in a microwave digestion system at 200 °C for 15 min. The digested samples were made up to an initial digest volume of 25 mL using ultrapure water (18Ω Millipore, MilliQ). They were further diluted 10-fold using ultrapure water immediately prior to being analysed for Hg by Inductively coupled plasma mass spectrometry (ICP-MS; Perkin Elmer DRCII) operating under standard conditions. Dry weight concentrations were then calculated based on the wet weight concentration of the analysed sample and the gravimetrically determined moisture content of a separate sub-sample. Samples of two certified reference materials, Dorm-3 (a fish protein CRM) and Dolt-5 (a dogfish liver CRM), both from National Research Council Canada, Ottawa, Canada, were run concurrently with the peregrine egg samples during the metals analysis.

Concentrations of elements in eggs are expressed as µg g^-1^ of dry weight (dw). The ICP-MS instrumental LoQ was calculated as 4.03 times the standard deviation of six replicate blank determinations. The mean tissue LoQ of Hg, Cd, Pb, and Se were 0.09, 0.002, 0.002, and 0.085 µg g^-1^ dw, respectively. The mean tissue LoQ of each metal ranged from 0.002 to 0.867 µg g^-1^ dw (SI Table 2). Concentrations of Cd in 19 eggs among 20 eggs were lower than LoQ (< LoQ), whereas no sample showed a concentration of Hg, Pb, and Se < LoQ. Given its high proportion of < LoQ (95% of all egg samples), Cd was removed from our data analysis. The percentage recovery from these CRMs ranged between 93 and 120%, depending on elements (SI Table 2).

### Analysis of carbon, nitrogen, and sulphur isotopes

Stable isotope (SI) analysis was carried out on the same 20 eggs in the stable isotope facility at the UKCEH Lancaster site and the Lancaster Environment Centre at Lancaster University. A 1 g sample of homogenised sub-sample egg contents was dried at 70 °C for 2 h prior to analysis. Samples were then weighed into tin capsules and combusted using an Eurovector elemental analyser. Resultant CO_2_ and N_2_ from combustion were analysed for δ^13^C and δ^15^N using a Micromass Isoprime isotope-ratio mass spectrometer (IRMS). The standard deviation for duplicate and quality control (QC) samples was less than 0.30‰ for δ^15^N and 0.35 for %N. For carbon, the standard deviation of QC samples was < 0.09‰ for δ^13^C and 2.25 for %C. Samples for analysing sulphur stable isotopes (δ^34^S) were combusted at 1120 °C on Tungstic Oxide in a Vario Pyrocube EA, and the δ^34^S isotopes were measured on an Isoprime100 IRMS. Repeat standards were run to an internal and external precision of < 0.2‰ standard deviations, while the difference for duplicate and QC samples was < 2.3% for δ^34^S.

### Data analysis

To deal with the pseudo-replication in our study (i.e., several eggs were collected from the same nests), we calculated the median value of the eggs for each of the three nests where several eggs had been collected, and the median value per nest (15 nests) was used for the following analyses.

We calculated the sum of all analysed PFAS (ΣPFAS), the sum of all analysed PFCAs (ΣPFCA), and the sum of all analysed PFSAs (ΣPFSA) for each nest. For the calculation of ΣPFAS, ΣPFCA, and ΣPFSA, values < LoQ of each compound were replaced by estimated values based on the regression on order statistic (ROS) when the given compound was detected from a large number of nests (> 10 nests) (Helsel 2012). Values < LoQ of compounds with a high proportion of non-detected values were set to 0 for the calculation. Differences in ΣPFAS, ΣPFCA, and ΣPFSA between the three sites were tested by the non-parametric Kruskal-Walls text, followed by multiple comparisons (Dunn’s test: non-parametric pairwise Mann–Whitney tests with Bonferroni correction). Differences in concentrations of each PFAS compound between the three sites were also tested by the same tests, when the given compound was detected from more than 10 nests. Differences in ESI and metal residues, including the ratio of Hg/Se, among the three regions were also tested in the same way. The ESI of our eggs was statistically compared with the ESI value at the pre-DDT level of 1.84 (Ratcliffe 1970), considered as the ESI value of non-contaminated eggs, by the *t*-test. Differences in each SI (i.e., δ^15^N, δ^13^C, δ^34^S) among counties were tested by the *F*-test followed by multiple comparisons (Tukey’s range test).

**Correlations across PFAS compounds:** Correlations between each pair of the PFAS compounds and metals, of which chemical was detected in more than 10 nests, were calculated and tested by non-parametric Pearson’s correlation index and test, respectively. PFAS and metal concentrations were logarithmically transformed to make their skewed distribution more normal.

**Relationship between contaminants and SI**: The relationship between PFAS residues and SI was analysed using a linear mixed-effects regression model (LMM) with sites as a random effect. The relationship was checked by PFAS compound: one LMM was built for each of the logarithmically transformed ΣPFAS, ΣPFCA, ΣPFSA, and PFAS compounds as the response variable, and the three SI were used as fixed effect variables. The model validation was carried out per model according to Zuur et al. (2009). Briefly, the full model (i.e., model containing all three SI as fixed effects) was compared between with and without the random effect (i.e., site) using Akaike Information Criteria (AIC) under Restricted Maximum Likelihood (REML) estimation to evaluate the pertinence of the random effect in modelling. We applied the likelihood ratio test (LR test) to check the significance of the random effect, and the model with a significantly lower AIC was selected and used to test the significance of the fixed effects. When the model with a random effect (i.e., LMM) was selected, we first measured the variance inflation factor (VIF) to detect multicollinearity among the fixed effects. A VIF of 1 indicates no collinearity, whereas higher VIF values suggest strong multicollinearity. A commonly used cutoff is VIF = 10, although even a VIF of 2 may cause non-significant parameter estimates (Zuur et al. 2010). The significance of SI was then checked by the LR test under Maximum Likelihood (ML) estimation. Non-significant variables were dropped from the model to build the final model containing statistically significant explanatory variables. We then measured two types of coefficients of determination (R^2^) for the final LMM under REML estimation: marginal R^2^ (R^2^_m_) representing the proportion of the total variance explained by the fixed effects and conditional R^2^ (R^2^_c_) representing the proportion of the variance explained by both fixed and random effects (Nakagawa et al. 2017). In contrast, when the model without a random effect was selected, we applied the *F*-test to the model (i.e., linear model) to examine the significance of variables and build the final model. The ordinal R^2^ of the final model was then calculated. We applied the same approach for the relationship between metal residues and SI. For this analysis, values < LoQ were replaced by values estimated by ROS (Helsel 2012), when applicable (i.e., the number of values < LoQ was small and from only one county).

The relationship between ESI and contaminant concentrations was also assessed in the same method. A LMM was built for ESI as the response variable, ΣPFAS was an explicative variable, and sites were integrated into model as a random effect. The model validation was carried out according to Zuur et al. (2009).

Statistical significance was assessed at α = 0.05 for all analyses. All statistical analyses were computed using the statistical software R (ver. 4.5.0) (Core Team 2025). LMM was performed by the function ‘*lme*’ from the package ‘*nlme*’. Marginal and conditional R^2^ were measured by the function ‘*r.squaredGLMM’* from the package ‘*MuMIn*’. ROS was conducted by the function ‘*cenros*’ from the package ‘*NADA*’.

## Results

### Descriptive statistics of PFAS concentrations in peregrine eggs

The detection rate differed among PFAS compounds (Table 1). PFNA, PFDA, PFdA, PFDoA, PFTrDA, PFTeDA, and PFOS in eggs were observed in 15 nests. PFOA was detected in 14 nests and was < LoQ in one nest in Cornwall. PFHxS was detected in 11 nests and was < LoQ in three nests in Cornwall and one nest in Lancashire. Some others were detected only in a few nests: PFHxA was detected only in one nest in Devon, PFDS was detected in five nests (four in Devon and one in Cornwall), and PFHxDA was detected in three nests in Devon. The other PFAS (PFBA, PFPeA, PFHpA, PFBS, PFODA, and PFOSA) were not detected in any nest. Except for PFHxA detected in one nest, only LC-PFAAs, particularly C8-C14 PFCAs and C6-C8 PFSAs, were detected from our samples.

**Table 1:** Statistical summary for PFAS in peregrine eggs from 15 nests. The minimum, arithmetic mean (Mean), median, and maximum of the concentrations (ng g wet weight) of each PFAS are shown. The distinction between PFCA/PFSA (PFAA type), number of carbon chain (C-chain length), number of specimens under the limit of quantification (No. < LoQ), contribution to summed PFAS and summed PFCA or PFSA (% of wet weight) are also given. The length class (short/long) is categorised according to OECD (2013). For PFOA and PFHxS, the mean value was calculated after imputation of the values < LoQ by the regression on order statistics (ROS) (Helsel 2012). FASAs: Perfluoroalkane Sulfonamides

	PFBA	PFPeA	PFHxA	PFHpA	PFOA	PFNA	PFDA	PFUdA	PFDoA
**PFAA type**	*PFCA*	*PFCA*	*PFCA*	*PFCA*	*PFCA*	*PFCA*	*PFCA*	*PFCA*	*PFCA*
**Length class**	*Short*	*Short*	*Short*	*Short*	*Long*	*Long*	*Long*	*Long*	*Long*
**C-chain length**	4	5	6	7	8	9	10	11	12
**No. < LoQ**	15	15	14	15	1	0	0	0	0
**Minimum**	-	-	< LoQ	-	< LoQ	0.170	0.280	0.173	0.312
**Mean**	-	-	-	-	0.178	0.489	0.996	0.864	1.488
**Median**	-	-	< LoQ	-	0.134	0.411	0.902	0.749	0.954
**Maximum**	-	-	0.05	-	0.928	0.970	4.574	1.881	4.742
**Contribution to ΣPFAS**	-	-	0.0-0.1%	-	0.0–2.0%	0.5–3.4%	1.4–4.6%	1.1-7.0%	1.4–7.7%
**Contribution to ΣPFCA/ΣPFSA**	-	-	0.0-0.6%	-	0.0-8.9%	3.3–13.2%	8.2–22.3%	7.7–16.6%	11.9–24.0%

	PFTrDA	PFTeDA	PFHxDA	PFODA	PFBS	PFHxS	PFOS	PFDS	PFOSA
**PFAA type**	*PFCA*	*PFCA*	*PFCA*	*PFCA*	*PFSA*	*PFSA*	*PFSA*	*PFSA*	*(FASAs)*
**Length class**	*Long*	*Long*	*Long*	*Long*	*Short*	*Long*	*Long*	*Long*	
**C-chain length**	13	14	16	18	4	6	8	10	
**No. < LoQ**	0	0	12	15	15	4	0	10	15
**Minimum**	0.415	0.261	< LoQ	-	-	< LoQ	5.222	< LoQ	-
**Mean**	1.868	1.645	-	-	-	0.180	25.997	-	-
**Median**	1.609	1.217	< LoQ	-	-	0.093	15.510	< LoQ	-
**Maximum**	4.178	4.236	0.104	-	-	0.754	77.888	0.287	-
**Contribution to ΣPFAS**	2.3–13.9%	2.1–6.9%	0.0-0.1%	-	-	0.0-1.1%	56.6–88.1%	0.0-0.4%	-
**Contribution to ΣPFCA/ΣPFSA**	19.7–32.7%	11.1–33.7%	0.0-0.8%	-	-	0.0-1.5%	98.1–100%	0.0-0.5%	-

The median value of ΣPFAS in eggs from 15 nests was 22.5 ng g^-1^ ww (range = 6.91–99.4 ng g^-1^ ww); the median of ΣPFCA and ΣPFSA were 6.06 (range = 1.68–20.6) and 15.6 ng g^-1^ ww (range = 5.23–78.9), respectively. Among PFAS compounds, PFOS showed the highest concentration (median = 15.5 ng g^-1^ ww; range = 5.22–77.9), followed by, but to a lesser extent, PFTrDA (median = 1.61 ng g^-1^ ww; range = 0.41–4.18), PFTeDA (median = 1.22 ng g^-1^ ww; range = 0.26–4.24), and PFDoA (median = 0.95 ng g^-1^ ww; range = 0.31–4.74). PFOS is therefore dominant in the PFAS profile (56.6–88.1% of contribution to ΣPFAS) and also in the PFSA profile (98.1–100% of contribution to ΣPFSA). PFHxS and PFDS marginally contribute to ΣPFSA (< 1.5% and < 0.5%, respectively). PFNA, PFDA, PFdA, PFDoA, PFTrDA, and PFTeDA more importantly contribute to ΣPFCA (3.3–33.4%) than PFHxA, PFOA and PFHxDA (0–8.9%).

### Comparison of PFAS concentrations between sites

**Fig. 1:**
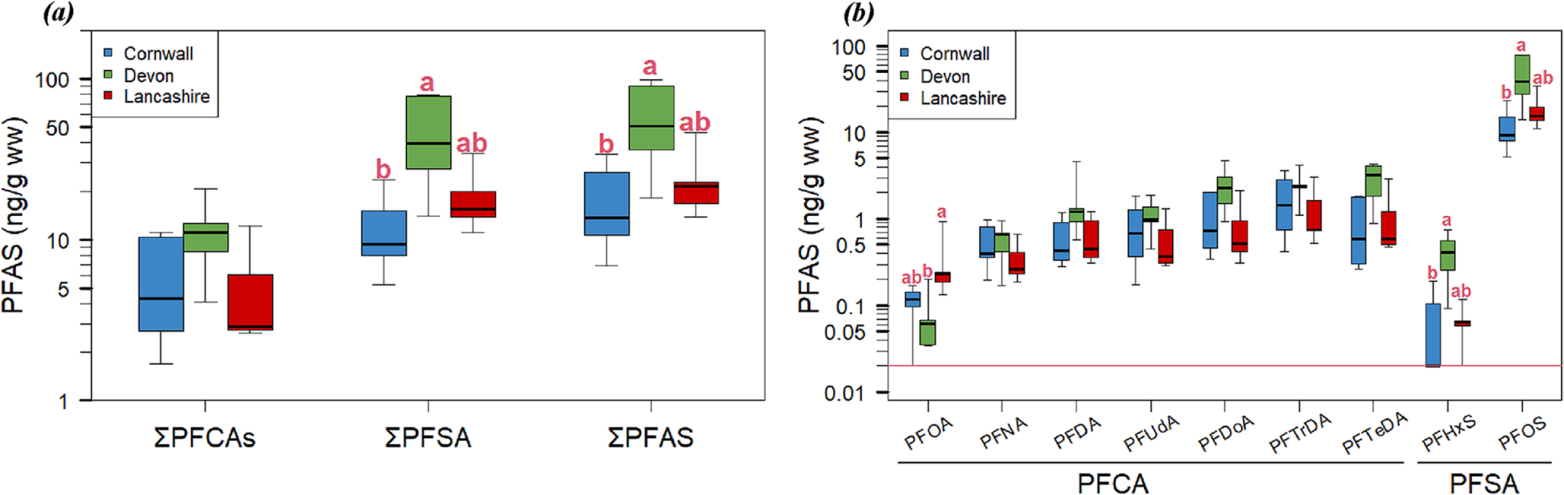
Boxplot representing concentrations of PFAS (ng g^-1^ wet weight) per county (Cornwall, Devon, and Lancashire). (a) Boxplot representing sums of PFAS, PFCA, and PFSA residues; (b) Boxplot representing concentrations of each of the PFAS compounds detected from more than 10 nests. Letters represent significant differences (p-value < 0.05) by Dunn’s non-parametric multiple comparisons. The red line represents the limit of quantification for PFOA and PFHxS

Concentrations of ΣPFAS significantly differed among the sites (Kruskal Wallis chi-squared = 6.02, df = 2, p-value = 0.049), and multiple comparisons indicated significantly higher ΣPFAS concentrations in nests in Devon (median = 50.5 ng g^-1^ ww) than in Cornwall (median = 13.6 ng g^-1^ ww) (Dunn’s test Z = -2.40, p-value adjusted with Bonferroni correction (adj.p-value) = 0.049) (Fig. 1a). Concentrations of ΣPFSA also significantly differed among the sites (KW chi-squared = 6.54, df = 2, p-value = 0.038) and were significantly higher in Devon (median = 39.6 ng g^-1^ ww) than in Cornwall (median = 9.34 ng g^-1^ ww) (Dunn’s test Z = -2.55, adj.p-value = 0.033). Concentrations of ΣPFCA did not significantly differ between the sites (Kruskal-Wallis chi-squared = 3.38, df = 2, p-value = 0.18).

**Fig. 2:**
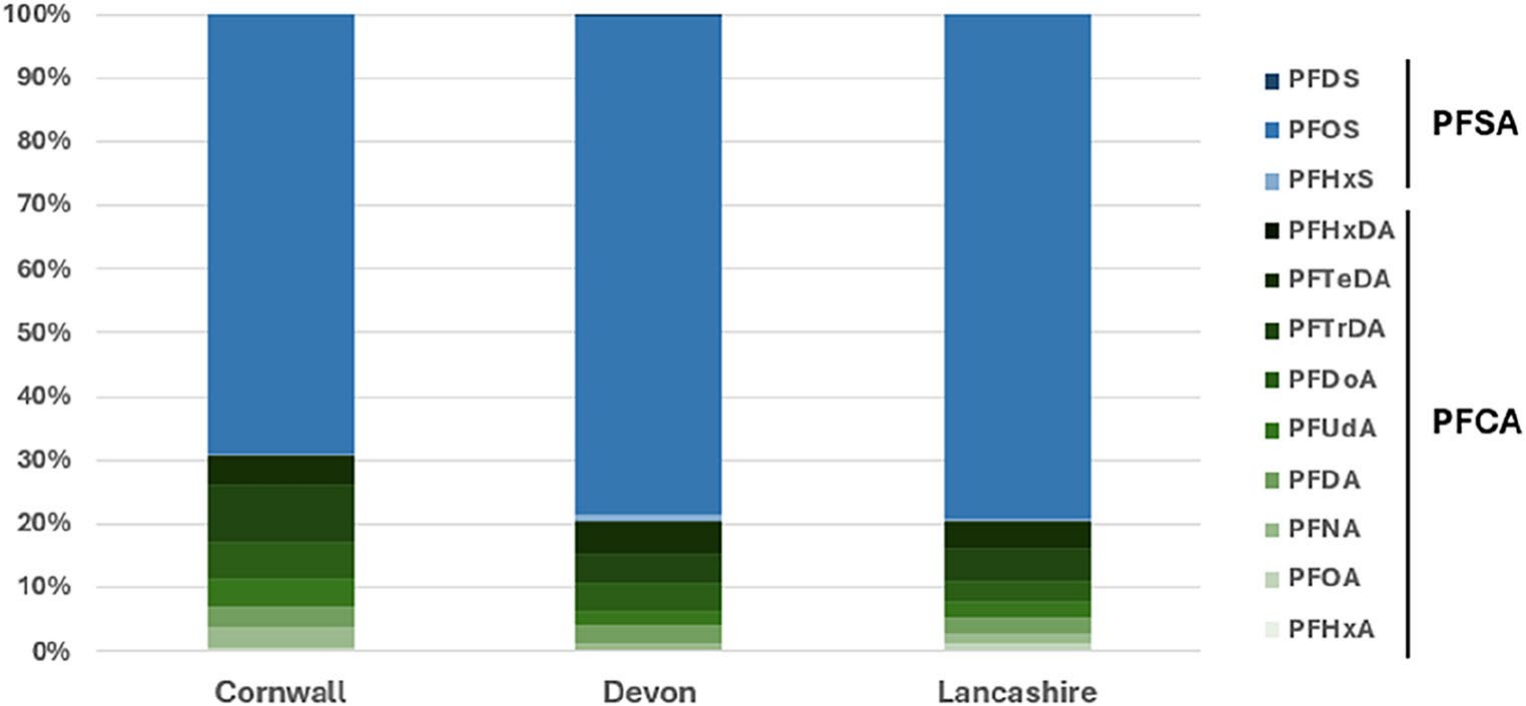
Composition profiles of PFAS compounds in peregrine eggs collected from Cornwall, Devon and Lancashire. The proportion was calculated using the mean PFAS residues per site

PFOA residues significantly differed between sites (KW chi-squared = 6.86, df = 2, p-value = 0.032) and were significantly higher in Lancashire (median = 0.23 ng g^-1^ ww) than in Devon (median = 0.061 ng g^-1^ ww) (Dunn’s test Z = -2.47, adj.p-value = 0.040) (Fig. 1b). PFTeDA residues also significantly differed between sites (Kruskal-Wallis Chi-squared = 6.18, df = 2, p-value = 0.046), but multiple comparisons showed no significant differences between any pairs of the sites. PFOS residues significantly differed between sites (Kruskal-Wallis Chi-squared = 6.54, df = 2, p-value = 0.038) and were significantly higher in Devon (median = 38.8 ng g^-1^ ww) than in Cornwall (median = 9.34 ng g^-1^ ww) (Dunn’s test Z = 2.54, adj.p-value = 0.033). There was no significant difference in the other PFAS compounds. The proportion of ΣPFCA in ΣPFAS was 30.8, 20.5, and 20.6% Cornwall, Devon, and Lancashire, respectively, while the proportion of ΣPFSA in ΣPFAS was 69.2, 79.5, and 79.4% (Fig. 2).

### Metal concentrations in peregrine eggs

The median of Hg residues observed in 15 nests was 0.34 µg g^-1^ dw (0.061 µg g^-1^ ww), and the range was 0.071–1.24 µg g^-1^ dw (0.014–0.182 µg g^-1^ ww). The median of Pb and Se residues were 0.044 and 2.60 µg g^-1^ dw, respectively (range = 0.01–0.22 and 1.84–3.84 µg g^-1^ dw). The median of the Hg/Se ratio was 0.145 (range = 0.033–0.323). Statistics for concentrations of elements are summarised in SI Table 2. There was no significant difference among sites in Hg, Pb, and Se residues, as well as the Hg/Se ratio (KW p-value > 0.05) (SI Fig. 2).

### ESI of peregrine eggs

The median of ESI was 1.94 (range = 1.80–2.10). The ESI values were significantly higher than the pre-DDT level: 1.84 (*t* = 4.04, df = 14, p-value = 0.01). The median per county was 2.02, 1.90, and 1.94 in Cornwall, Devon, and Lancashire, respectively. There was no significant difference in ESI values among counties (KW chi-squared = 0.74, df = 2, p-value = 0.69). The index values in Lancaster were significantly higher than the pre-DDT level (*t* = 2.92, df = 4, p-value = 0.043), while the values in Cornwall and Devon did not significantly differ from the pre-DDT level (t = 2.01, df = 4, p-value > 0.05) (SI Fig. 3).

### SI of peregrine eggs

**Fig. 3:**
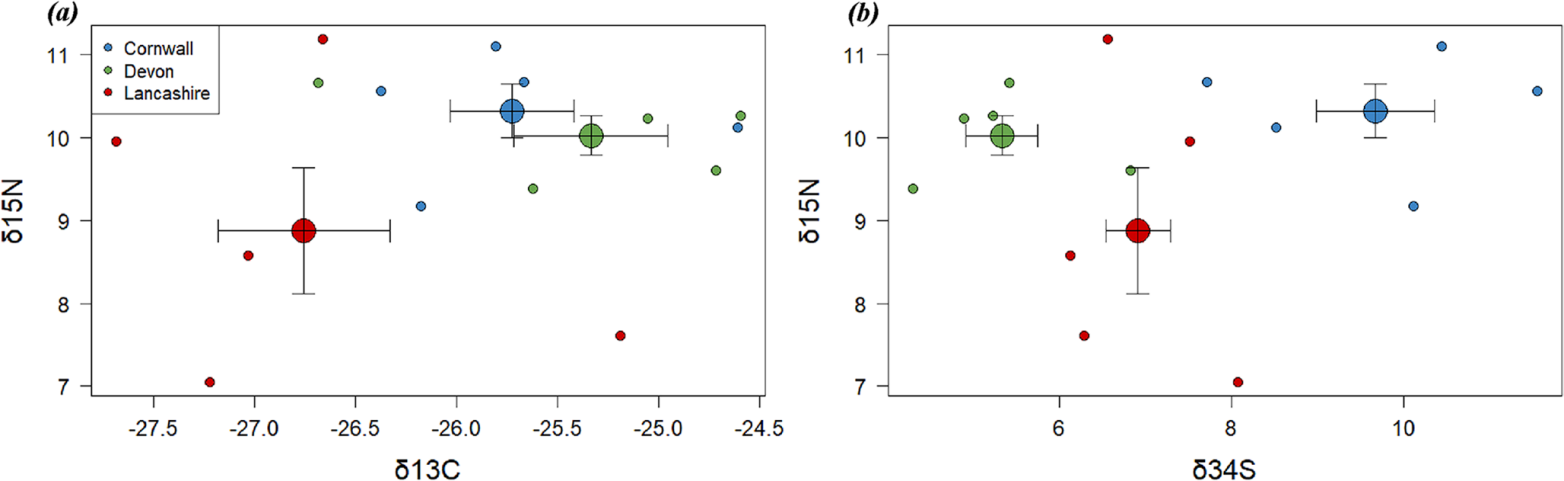
Scatter plots representing the relationships between δ15N and δ13C (a) and between δ15N and δ34S (b) in peregrine eggs. Each small point represents stable isotope values of each egg. Counties are distinguished by colours (blue: Cornwall; green: Devon; red: Lancashire). Larger points and bars represent the mean value and the standard error of the mean by county

The mean values of SI were 9.74 (standard deviation = 1.21), -25.9 (sd = 0.99), and 7.31 (sd = 2.14) for δ^15^N, δ^13^C, and δ^34^S, respectively. The mean of δ^15^N was 10.32 (sd = 0.32), 10.02 (sd = 0.23), and 8.87 (sd = 0.76) in Cornwall, Devon, and Lancashire, respective, whereas the mean of δ^13^C was − 25.73 (sd = 0.31), -25.34 (sd = 0.38), and − 26.76 (sd = 0.43), and the mean of δ^34^S was 9.67 (sd = 0.69), 5.34 (sd = 0.42), and 6.92 (sd = 0.38) in the three sites, respectively (Fig. 3). Only δ^34^S in eggs significantly differed among sites (*F* = 18.3, p-value < 0.001), but not δ^13^C (*F* = 3.85, p-value = 0.051) and δ^15^N (*F* = 2.37, p-value = 0.14).

### Correlations between PFAS and metals

Except for PFOA, all PFAS compounds showed significantly positive correlations with each other (Pearson’s correlation index *r* ranges 0.69–0.96; SI Fig. 4). PFOA was significantly correlated with none of the other PFAS compounds. Mercury showed significantly positive correlations with all PFAS compounds (*r* ranges 0.58–0.89), except for PFOA. Selenium was significantly and positively correlated with Hg (*r* = 0.52) but not with any PFAS.

### Relationships between SI and PFAS/metals

**Fig. 4:**
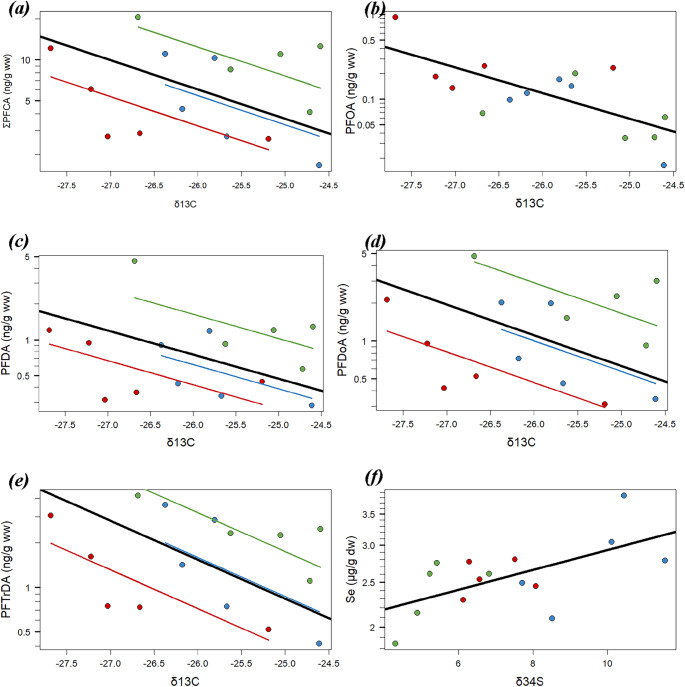
Concentrations of ΣPFCA (a), PFOA (b), PFDA (c), PFDoA (d). PFTrDA (e), and selenium (f) in peregrine eggs collected in the UK in relation to stable isotope (a – e: δ^13^C; f: δ^13^C). Each point represents a logarithmically transformed concentration of the given PFAS of an individual egg (ng g^-1^ wet weight). The thick black line represents the whole trend of the relationship from the linear mixed effect regression model (a, c – e) or the trend of the liner regression model (b and f). The thin coloured line represents the trend of the relationship in each county (blue: Cornwall; green: Devon; red: Lancashire)

ROS was applied to the PFOA residue data that included one < LoQ value. In contrast, ROS could not be applied to the PFHxS data because several values < LoQ were observed across different counties, preventing the assignment of appropriate estimated values to each county. During our model validation process, no collinearity was observed among SI (VIF < 2). There was no significant relationship between any of the three SI and ΣPFAS or ΣPFSA. None of the PFSA compounds showed a significant trend along with any of the SI. In contrast, the final model for ΣPFCA, containing sites as a random effect (LR = 5.06, p-value = 0.024), showed a significantly decreasing trend along with δ^13^C (LR between the final and null model = 4.36, p-value = 0.036; R^2^_m_ = 0.234, R^2^_c_ = 0.751; Fig. 4a). The final model for PFOA does not contain a significant random effect but showed a significantly decreasing trend along with δ^13^C (*F* = 12.9, p-value = 0.003; R^2^ = 0.498; Fig. 4b). Significantly decreasing trends along with δ^13^C were also observed in models for other PFCAs: PFDA (LR = 3.90, p-value = 0.048; R^2^_m_ = 0.203, R^2^_c_ = 0.730; Fig. 4c), PFDoA (LR = 4.92, p-value = 0.027; R^2^_m_ = 0.201, R^2^_c_ = 0.797; Fig. 4d), and PFTrDA (LR = 5.88, p-value = 0.015; R^2^_m_ = 0.298, R^2^_c_ = 0.804; Fig. 4e). In contrast, no significant relationship with SI was observed in PFNA, PFUdA, and PFTeDA.

There was no significant relationship between any of the SI and Hg or Pb. However, the final model for Se, which contained no random effect, showed a significantly increasing trend of Se along with δ34S (*F* = 7.65, p-value = 0.016, R^2^ = 0.371; Fig. 4f).

The modelling for ESI showed no significant relationship with ΣPFAS (p-value > 0.05).

## Discussion

### PFAS residues in eggs

PFAS residues in our peregrine eggs from study areas were mainly composed of LC-PFAAs, predominantly C8–C14 PFCAs and C6–C8 PFSAs. Particularly, we observed a high proportion of PFOS in the PFAS profile. A similar PFAS profile has been reported in previous studies on terrestrial raptor eggs. There were no detectable SC-PFAAs (PFBS, PFHxA, PFHpA) and PFOSA in Swedish peregrine eggs collected in 2006 (Holmström et al. 2010). Likewise, SC-PFAAs were not detected in eggs of tawny owl (*Strix aluco*) from Norway in 1986–2009 (Ahrens et al. 2011a) and in eggs of tawny owl and of common kestrel (*Falco tinnunculus*) from Sweden in 2014 (Eriksson et al. 2016). The dominance of PFOS, as well as high concentrations of some LC-PFCAs like PFDA or PFDoA, have been unanimously reported, but the magnitude of PFAS residues varies among studies. For example, the Swedish peregrine eggs in 2006 (Holmström et al. 2010) showed a mean PFOS concentration of 83 ng g^-1^ ww, which is much higher than the mean of PFOS in our eggs (24.4 ng g^-1^ ww). In contrast, Eriksson et al. (2016) reported 7.9 and 3.8 ng g^-1^ ww as the median of PFOS in eggs of Swedish tawny owls and kestrels in 2014, respectively. Concentrations of the other PFAS compounds also widely vary across these studies.

Exposure to PFAS reported in the literature considerably varies across different areas, regions, and countries. As with many other chemical contaminants, environmental PFAS concentrations are drastically reduced from their sources. In the study of Groffen et al. (2017), the median of PFOS and PFOA in eggs of great tits (*Parus major*) reduced from a range of 10,380 and 19.8 ng g^-1^ ww close to a fluorochemical plant 3M in Belgium to 9.4 and 0.3 ng g^-1^ ww in about 70 km from the plant, respectively. A reduction in PFAS exposure with increasing distance from a PFAS plant was also observed in eggs of northern lapwings (*Vanellus vanellus*) in Belgium (Lopez-Antia et al. 2017). Likewise, various PFAS compounds in plasmas of white-tailed eagle (*Haliaeetus albicilla*) nestlings significantly reduced from the airport, where fluorosurfactants containing AFFFs were possibly used (Jouanneau et al. 2020). In contrast, long-distance transport of PFAS from sources has also been proven, as PFAS can reach remote areas such as the Arctic marine ecosystem (Blévin et al. 2017; Houde et al. 2011; Letcher et al. 2010).

PFAS exposure in our study also varied among our UK study areas. Higher PFOS and PFHxS concentrations in eggs from Devon may be due to the nests’ location. High PFSA burdens are linked to anthropogenic sources releasing PFAS compounds into the environment, such as landfills or the use of PFAS-based products (Ahrens et al. 2011b; Environment Agency 2024; Hamid et al. 2018). Compared to the other areas, the nests in Devon are located in urban areas around where there are international Exeter and Bournemouth airports, which may explain high PFSA burdens (Ahrens et al. 2015; Kwadijk et al. 2014). Meanwhile, significantly higher egg PFOA residues in Lancashire concur with the findings of O’Rourke et al. (2022): PFOA concentrations in the livers of UK Eurasian otters significantly decreased from a fluoropolymer factory located in Lancashire. Although we found no detailed data on PFAS emissions from this factory, it is likely that other PFAS compounds, including other PFCAs, were released from the factory (Liang et al. 2024). However, we found no significant difference in other PFCA residues across the three sites, suggesting that the source location may not be the only factor explaining wildlife exposure.

The differences in transfer routes among ecosystems and species also contribute to the spatial variation in PFAS residues in wildlife. There are unquestionable differences in the PFAS exposure and response among marine, freshwater, and terrestrial biota. The marine environment is the major sink of PFAS, particularly SC-PFAAs due to their water solubility (Ahrens and Bundschuh 2014; Health and Safety Executive, 2023; Johansson et al. 2019), while the enrichment of PFAS compounds in organisms depends on not only their trophic position but also retention and elimination features; for example, compounds with low K_OW_ and high K_OA_ like PFOA or PFOS could be eliminated from the livers of aquatic animals through their gills but hardly eliminated from lung-breathing animals (Chen et al. 2021; Kelly et al. 2007). As a result, differences in habitats and diets lead to interspecific variation in PFAS burdens, as reported in the studies of Eriksson et al. (2016); Guruge et al. (2011); Ahrens et al. (2011a).

Our peregrine eggs showed a large variation in SI across counties. Although not significantly different from the other sites, δ^13^C in Lancaster was lower than the other sites, whereas δ^34^S in Cornwall was significantly higher than the other sites. As δ^13^C and δ^34^S are spatial markers (Inger and Bearhop 2008), our results indicate that peregrines from the three different areas had different feeding habitats. Peregrines in Lancashire are supposed to be more terrestrial than the others, while those in Cornwall are more marine. Interestingly, concentrations of PFOS, PFOA, PFNA, and PFTrDA in eggs were significantly correlated with δ^13^C, but not by δ^34^S or δ^15^N. In the study of Jones et al. (2024), which analysed stable isotopes of kestrel nestling feathers collected from East Dorset, differences in δ13C signatures reflect differences in their diet, independent of sampling locations within their study area of 10 km^2^. Our results suggest that PFAS burdens in our eggs might be associated with differences in food inputs, such as differences in foraging between terrestrial and nearshore environments. Monclús et al. (2022) also observed a significant relationship between SI and PFAS burdens: concentrations of PFAS in feathers of Norwegian Eurasian eagle-owls (*Bubo bubo*) were significantly higher with high δ^13^C values in feathers. These authors concluded that birds feeding on a ^13^C-enriched diet, which may reflect marine carbon input from seabirds and fish (Kelly 2000), increased PFAS residues in their eagle-owls. In our study, however, specific PFCA residues were negatively correlated with δ^13^C values, suggesting that high PFCA residues might be associated with terrestrial carbon input. In contrast, Se was positively correlated with δ^34^S values that are also associated with food items from different ecosystems (i.e., marine, freshwater, and terrestrial diets) (Wiley et al. 2017). However, although details of the diet of UK peregrine differ across regions, such as feeding on coastal or seabirds in coastal areas, UK peregrines mainly feed on terrestrial pigeons (Ratcliffe 1993; Sale 2016). Moreover, our analysis demonstrates that, except for PFOA, PFCA residues in eggs were more importantly explained by the random effect of counties than by δ^13^C. Although a relationship between PFCA residues and δ^13^C was observed across our three areas, the sources of PFCAs associated with low δ^13^C values may differ among the areas. As we have no stable isotope data for peregrine’s food items, it is not possible to conclude which foods from which ecosystem may affect PFAS in birds. Identifying the foods most likely to contribute to PFAS burdens is still a question for further studies.

Meanwhile, it is also important to consider that exposure to PFAS may be direct (i.e., exposure to the given compound transported from sources) or indirect (i.e., exposure to the given compound due to the degradation of precursors). Many studies have reported a particular pattern of high concentrations of odd-numbered carbon PFCAs and low concentrations of even- numbered carbon PFCAs in organisms’ tissues (Ahrens et al. 2011a; Faxneld et al. 2016; Holmström et al. 2010; Holmström and Berger 2008; Park et al. 2021; Pereira et al. 2021; Sun et al. 2019; Verreault et al. 2005; Yoo et al. 2008). Odd- and even-numbered carbon patterns are from PFAS manufacturing processes (Buck et al. 2011; Moody and Field 2000). Electrochemical fluorination, a technology leading to the replacement of all the H atoms of an organic raw material by F atoms, generates even- and odd-number perfluorocarbons, whereas telomerisation in which perfluoroalkyl iodide (telogen) reacts with tetrafluoroethylene (taxogen) tends to produce mixtures of even chain lengths. Moreover, odd-even patterns in the environment could be attributed to significant inputs from atmospheric sources. Even-numbered fluorotelomer-based precursor compounds, like FTOHs, are transported over long distances in the atmosphere and degrade to odd- and even-numbered PFCAs, which results in high concentrations of odd-numbered PFCAs due to their bioaccumulation capacity (Armitage et al. 2009; Ellis et al. 2004). Our peregrine eggs did not show such an odd-even pattern; odd- and even-numbered carbon PFCAs showed similar concentrations in our results, as observed in the studies on Swedish osprey’s eggs (Eriksson et al. 2016) or UK otter’s livers (O’Rourke et al. 2022). These authors concluded that direct exposure from local sources might be more important for freshwater predators than indirect exposure from atmospheric degraded precursors. In line with these studies, we hypothesise that exposure of our peregrine populations to PFAS may be from local sources rather than from the degradation of precursors. On the other hand, PFCAs longer than eight-chain are more likely transferred from mother to eggs than ≤8C PFCAs, such as PFOA (Gebbink and Letcher 2012; Holmström and Berger 2008; Nordén et al. 2013; Verreault et al. 2005), which may be another potential explanation for the non-significant correlation of PFOA with the other PFCAs in our eggs. However, such maternal transfer depends on species (Gebbink and Letcher 2012), and it is still unknown how odd-even patterns are modulated by tissue-specific PFAS accumulation. The distinction between direct and indirect sources of exposure needs further studies on the distribution of PFAS within bodies and eggs.

### Possible impact of PFAS or metals on peregrine populations in West England

Our study showed no statistically significant relationship between ESI and PFAS or metals. Moreover, the eggshell index value of our sample was similar to the pre-DDT level of 1.84 (Ratcliffe 1970), or even higher in Lancashire. We have no explanation for the thicker eggshell in the samples from Lancashire compared to those from the other areas. Some PFAS compounds might positively influence eggshell formation, as PFOS exposure has been shown to stimulate COX-2 expression in human cells (Liao et al. 2012) and in embryos of marine fish (Huang et al. 2011). However, PFAS exposure was not significantly higher in Lancashire, except for PFOA. These results indicate that PFAS exposure neither positively nor negatively contributes to eggshell formation. Given that our macroscopic inspection showed no visible abnormality in embryo development, the decline in the Cornwall peregrine population is not explained by breeding failure due to eggshell thinning.

Nonetheless, it is still unclear whether PFAS contribute to the peregrine population decline, like Hg which is assumed to be implicated in the brood-size reduction in UK peregrine populations but not in eggshell thinning (Newton et al. 1989). Various adverse effects of PFAS have been reported, such as immunotoxicity, hepatotoxicity, hormonal disruption, and impairment of reproductive success (Lau et al. 2004; Letcher et al. 2010; Nøst et al. 2012). Groffen et al. (2019) showed that high concentrations of certain PFAS, such as PFOS and LC-PFCAs, might be associated with a reduced hatching success of great tit nests. A Lowest-Observable-Adverse-Effect (LOAEL) for PFOS in bird eggs was estimated as 100 ng g^-1^, based on reduced hatchability in white leghorn chickens (*Gallus gallus domesticus*) after a PFOS injection to the air cell of the egg (Molina et al. 2006). Moreover, the avian predicted no effect concentration (PNEC) for PFOS was estimated as 1000 ng/mL in the egg yolk, based on reproductive effects in the bobwhite quail (*Colinus virginianus*) and the mallard (*Anas platyrhynchos*) dietarily exposed to PFOS (Newsted et al. 2005). This value is equivalent to 290 ng g^-1^ in a whole egg, assuming that an egg is composed of 29% of yolk and 71% of albumen in wet weight, as measured in herring gull (*Larus argentatus*) eggs (Gebbink and Letcher 2010), and that the yolk has 1.0 g/mL specific gravity. Although exposure methods and species may cause differences in LOAEL or PNEC values, none of our eggs had PFOS concentrations exceeding these two reference values. We therefore conclude that PFOS exposure might not affect peregrine population decline based on the threshold values discussed above. Moreover, the concentrations of heavy metals in our samples were lower than the threshold values reported in the literature. Newton et al. (1989) considered 0.2 µg g^-1^ ww of Hg in UK peregrine eggs (equivalent to 1 µg g^-1^ dw with the average moisture content of our eggs) as a concentration associated with no adverse effect on the reproduction. Although Cd affects eggshell thickness and bird reproduction (Wayland and Scheuhammer 2011), most of our samples showed Cd in egg < LoQ. Monitoring Pb in eggs is rare because maternal transfer of this metal to eggs is low (Furness 1993; Monclús et al. 2020). The positive correlation between Se and δ34S indicates different sources of Se, but Se concentrations in our samples were within a range of the global background level: mean background Se concentrations are typically 1.5–2.5 µg g^-1^ dw for both marine and terrestrial bird eggs, and maximums of background levels for individual eggs are < 5 µg g^-1^ dw (Ohlendorf and Heinz 2011). In addition, all egg samples showed a Hg/Se ratio < 1. Concentrations of the other non-essential metals, such as strontium, were also low in our samples and are not thought to be associated with any adverse effects. We therefore propose that there may be no or low risk of harmful effects of heavy metals on peregrines at these study sites.

However, some individual eggs had a PFOS concentration close to the PNEC value or a Hg concentration above the no adverse effect value. Moreover, some PFAS may be more toxic than PFOS, although data on the toxicity reference value for avian species remain limited. For example, Bil et al. (2021) demonstrated differences in potency across PFAS compounds for liver endpoints in male rats using the relative potency factor methodology, showing higher potency for C9-12 LC-PFCAs than for PFOA. The mechanisms underlying the adverse effects of PFAS on birds are still poorly understood, but they may induce different effects from those of lipophilic contaminants. Unlike other persistent organic pollutants, PFAS are accumulated in certain protein-rich tissues, such as blood and liver (Giesy and Kannan 2001; Sletten et al. 2016), because of their affinity to specific proteins such as albumin and fatty acid binding portions (Jones et al. 2003; Ng and Hungerbühler 2013). Phospholipids are also a determinant factor of the internal tissue distribution and bioaccumulation of LC-PFAS (Armitage et al. 2012; Dassuncao et al. 2019). It is still obscure whether other PFAS than PFOS might affect the breeding success of peregrines and their populations in Cornwall. We need further studies on the adverse effects of PFAS on wild bird populations.

## Conclusion

In this study, we measured PFAS and metal concentrations in peregrine eggs from West England counties and compared these values with SI and ESI. Concentrations of metals in eggs were low and did not significantly differ among counties. Certain PFAS in eggs, like PFOA and PFOS, significantly differed among counties, and these differences might be due to PFAS in local environmental sources rather than PFAS from long-distance sources transported over in the atmosphere, given similar concentrations between odd- and even-numbered PFCAs. The data on SI also showed that exposure of peregrines to PFAS might be significantly influenced by variation in local sources, but not by their trophic levels, in our study areas. Meanwhile, we did not find any significant relationship between PFAS in eggs and ESI. With the result of the ESI values of our eggs that were similar or even higher than the pre-DDT level, we propose that the current decline in the peregrine population on the North Cornish coast is unlikely to be, at least, due to eggshell thinning. However, knowledge of the adverse effects of PFAS on bird populations is limited, especially for those other than PFOS. Moreover, potential interactions among diverse types of chemical contaminants remain unclear. Further studies are therefore needed to fill such knowledge gaps.

## Supplementary Information

Below is the link to the electronic supplementary material.

Supplementary Material

## Data Availability

All data will be made available upon request.
