# Supplementary material for: Per- and polyfluoroalkyl substances (PFAS) and heavy metals in the egg of peregrine falcon (Falco peregrinus) populations in West England, United Kingdom
Source: Ecotoxicology. 2026 Apr 11;35(5):96. doi: 10.1007/s10646-026-03076-x (PMC13070063; doi:10.1007/s10646-026-03076-x)
Supplement: Supplementary file 1 — Supplementary Material [file 10646_2026_3076_MOESM1_ESM.pdf]

Supplementary Information for:

Per- and polyfluoroalkyl substances (PFAS) in the egg of peregrine falcon (*Falco peregrinus*) populations in West England, United Kingdom

(Published in *Ecotoxicology*)

Shinji Ozaki[1], Jacqueline S. Chaplow[1], Beverley Dodd[1], Helen Grant[1], M. Glória Pereira[1], Elaine Potter[1], Richard G. Sale[2], Darren Sleep[1], Sarah Thacker[1], Steve J. Watson[3], Lee A. Walker[1], and Suzane M. Qassim[4]*

1 UK Centre for Ecology & Hydrology, Lancaster Environment Centre, Library Avenue, Bailrigg, Lancaster, LA1 4AP, United Kingdom

2 Independent researcher, Coberley, GL53 9QY, United Kingdom

3 South-West Peregrine Group, Old Builders Arms, Randalls Green, Chalford Hill, Stroud, Gloucestershire, GL6 8EF, United Kingdom

4 Natural England, 4th Floor, Eastleigh House, Upper Market Street, Eastleigh, SO50 9YN, United Kingdom

* Corresponding author: Shinji Ozaki
E-mail address: ShiOza@ceh.ac.uk

Table of Contents:

**Supplementary Information Fig. 1**	Page S1
**Supplementary Information Fig. 2**	Page S2
**Supplementary Information Fig. 3**	Page S3
**Supplementary Information Fig. 4**	Page S4
**Supplementary Information Table 1**	Page S5
**Supplementary Information Table 2**	Page S6
**Reference**	Page S7

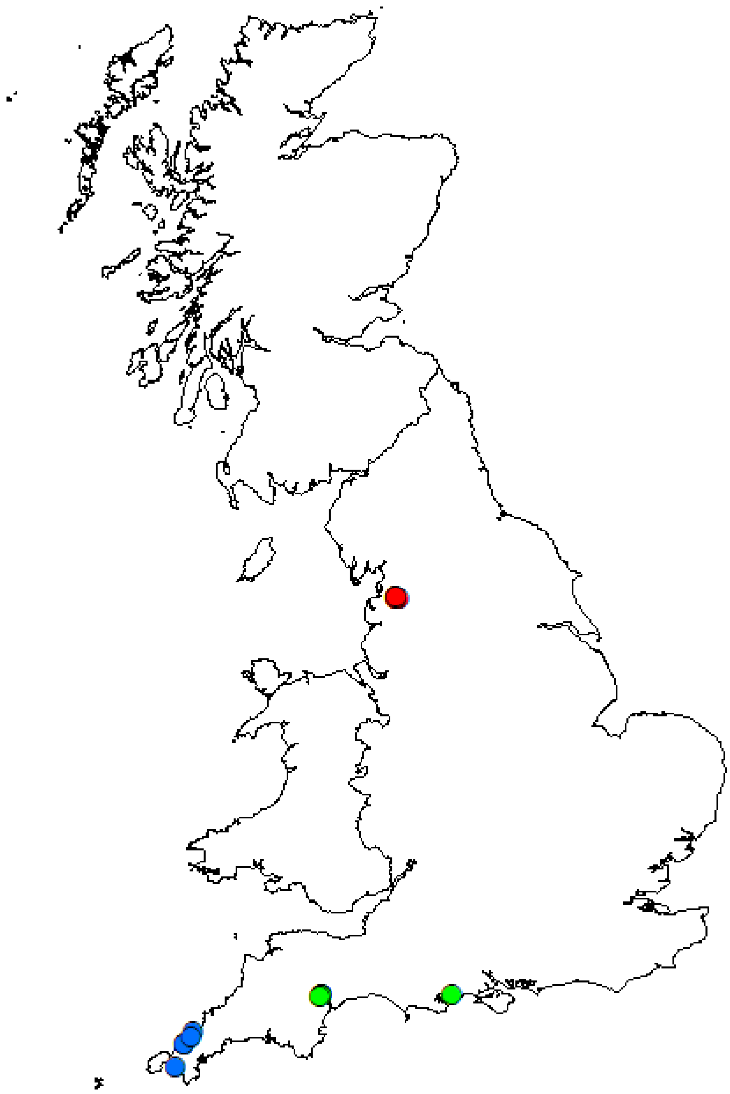

Supplementary Information Fig 1 *Map representing the location of the nests from which egg samples were collected. The three sites are distinguished by colours (blue: Cornwall; green: Devon & Dorset; red: Lancashire).*

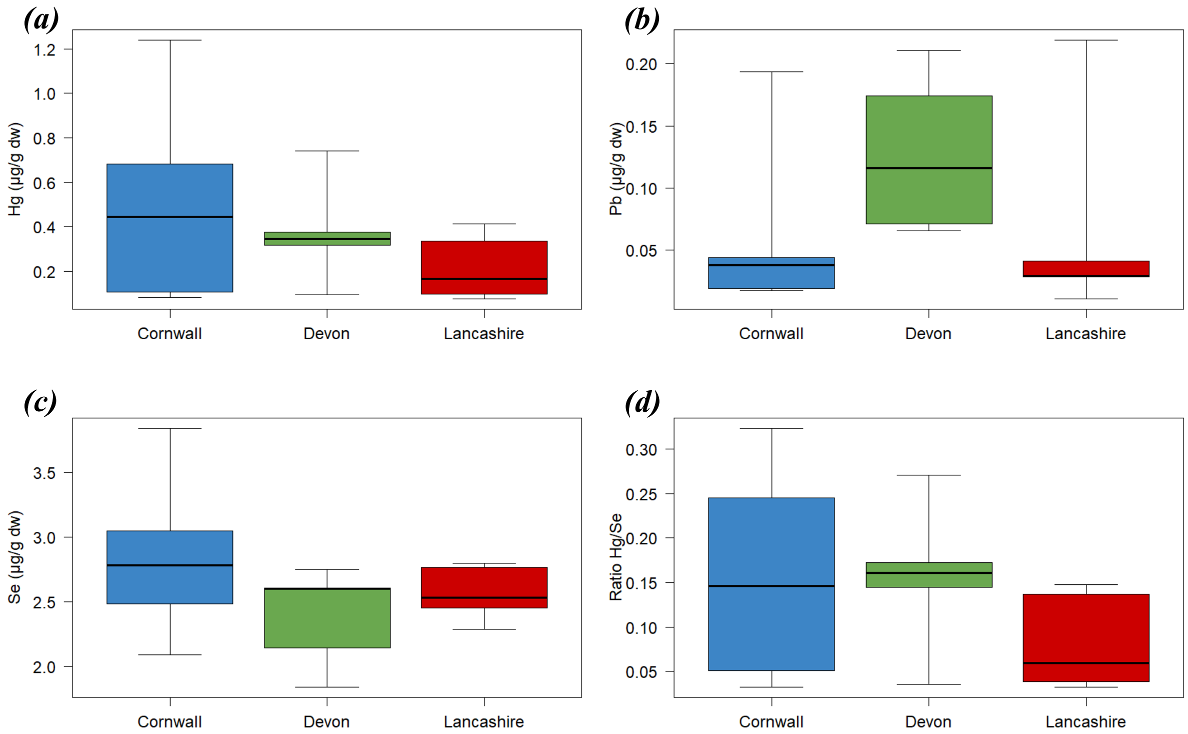

Supplementary Information Fig. 2 *Graphical representations of concentrations of mercury (a), lead (b), selenium (c) and the ratio Hg/Se (d) in peregrine eggs (µg g^{-1} dry weight) per county (Cornwall, Devon, and Lancashire). No significant differences in concentrations of Hg, Pb, Se, and the ratio Hg/Se among counties were demonstrated by the Kruskal-Wallis test.*

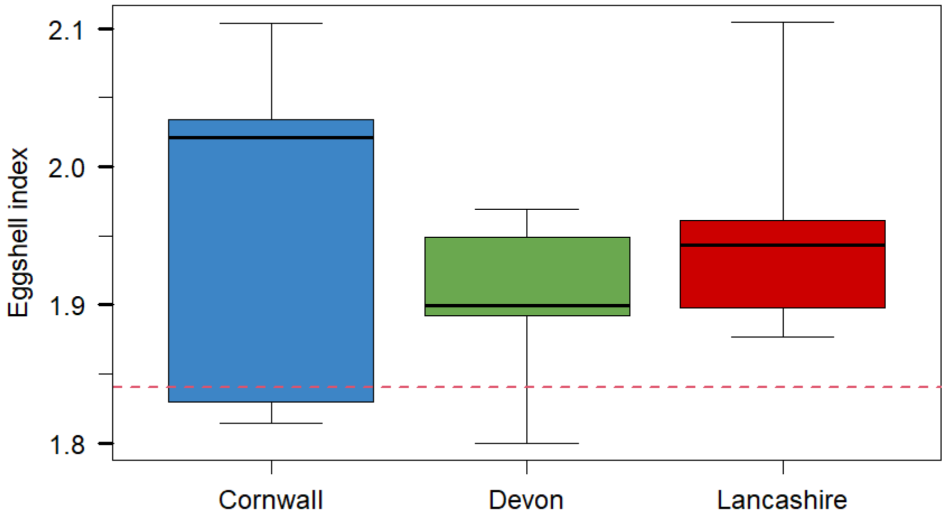

Supplementary Information Fig. 3 *Graphical representation of eggshell index values per county (Cornwall, Devon, and Lancashire). The red dashed line represents the eggshell index value of the UK peregrine at the pre-DDT level of 1.84 (Ratcliffe, 1970). No significant difference in eggshell index values among counties was demonstrated by the Kruskal-Wallis test. Only eggshell index values in Lancashire were significantly higher (t-test; p-value = 0.043) than the pre-DDT level of 1.84.*

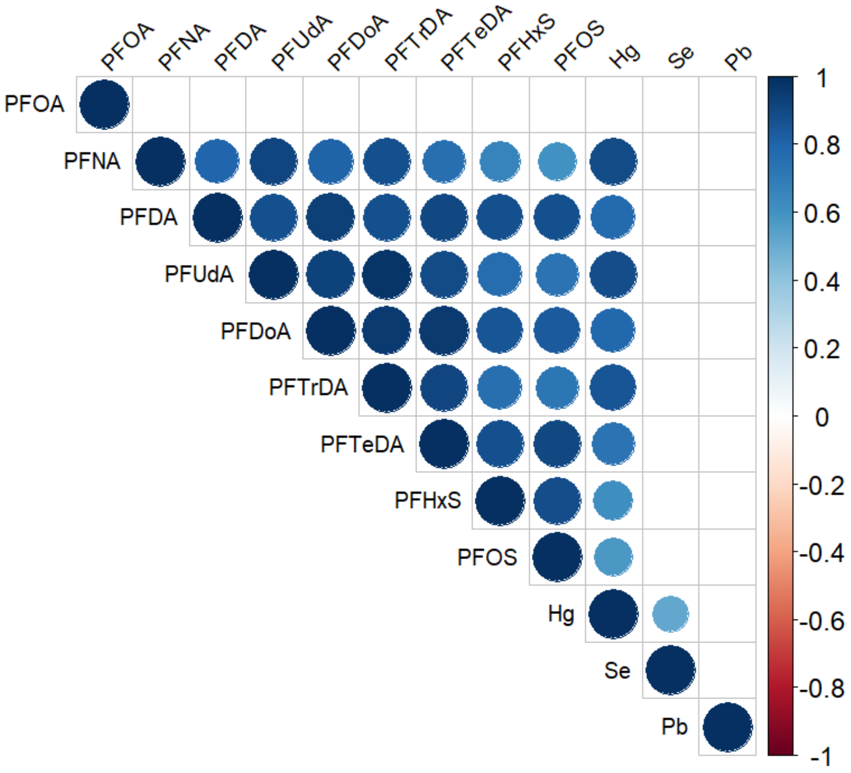

Supplementary Information Fig. 4 *Significant correlations between each pair of PFAS compounds and metals (mercury, selenium, and lead). The size and depth of the circles' colour indicate Pearson's correlation coefficients. Blue circles indicate significant and positive correlations based on Pearson's correlation coefficient test. No significant negative correlation was observed in the data.*

Supporting Information Table 1 *Name, abbreviation, CAS number and limit of quantification value (LoQ; ng g^{-1} wet weight), and recovery rate of each PFAS compound measured in this study. The recovery rate is given only for the ^{13}C labelled standards (PFBA, PFHxA, PFHxS, PFOA, PFNA, PFOS, PFDA, PFUdA, PFDOA, and PFTeDA)*

**PFAS**		**CAS Number**	**LoQ** (*ng/g ww*)	**Recovery rate**	
**Abbreviation**	**Name**			**Mean**	**Range**
PFBA	Perfluorobutanoate	456-22-4	0.08	81%	73-93%
PFPeA	Perfluoropentanoate	5989-64-0	0.08	-	-
PFBS	Perfluorobutane sulfonate	375-73-5	0.02	-	-
PFHxA	Perfluorohexanoate	307-24-4	0.02	81%	70-89%
PFHpA	Perfluoroheptanoate	375-85-9	0.02	-	-
PFHxS	Perfluorohexane sulfonate	355-46-4	0.02	83%	71-99%
PFOA	Perfluorooctanoate	335-93-3	0.02	82%	69-97%
PFNA	Perfluorononanoate	444-03-1	0.02	83%	73-93%
PFOS	Perfluorooctane sulfonate	2795-39-3	0.02	88%	73-98%
PFDA	Perfluorodecanoate	335-76-2	0.02	85%	71-111%
PFUdA	Perfluoroundecanoate	2058-94-8	0.05	94%	63-120%
PFDS	Perfluorodecane sulfonate	335-77-3	0.02	-	-
PFDoA	Perfluorododecanoate	307-55-1	0.02	84%	61-118%
PFTrDA	Perfluorotridecanoate	72629-94-8	0.02	-	-
PFTeDA	Perfluorotetradecanoate	376-06-7	0.02	78%	60-107%
PFHxDA	perfluorohexadecanoate	67905-19-5	0.02	-	-
PFODA	Perfluorooctadecanoic acid	16517-11-6	0.05	-	-
PFOSA	Perfluorooctane sulfonamide	754-91-6	0.02	-	-

Supporting Information Table 2 *Statistical summary for elements in peregrine eggs from 15 nests. The minimum, arithmetic mean (Mean), median, and maximum of the concentrations of each element (µg g^{-1} dry weight) are shown. The limit of quantification (LoQ; µg g^{-1} dry weight), number of the samples (nests) under LoQ (No. <LoQ), and recovery rates compared to the two certified reference materials (Dorm-3: a fish protein CRM; Dolt-5: a dogfish liver CRM) of 13 elements measured by ICP-MS in this study are also given. The mean value of chromium is calculated after imputation of the values <LoQ by the regression on order statistics (ROS) (Helsel, 2012). Mercury concentrations are also mentioned in µg g^{-1} wet weight. Statistics on the Hg/Se ratio are also included.*

		**Chromium Cr**	**Iron Fe**	**Cobalt Co**	**Nickel Ni**	**Copper Cu**	**Zinc Zn**	**Arsenic As**
**LoQ**	**Value**	0.018	0.867	0.002	0.002	0.018	0.867	0.006
	**No. <LoQ**	2	0	0	0	0	0	0
**Statistics**	**Minimum**	<LoQ	58.32	0.006	0.012	1.560	36.4	0.035
	**Mean**	0.063	79.86	0.012	0.082	2.402	48.3	0.122
	**Median**	0.045	74.23	0.010	0.049	2.271	47.7	0.090
	**Maximum**	0.265	109.22	0.021	0.264	3.844	60.7	0.610
**Recovery rate**	**DORM-3**	96.5	98.1	-	109.3	106.5	110.8	104
	**DOLT-5**	-	100.3	107.4	-	100.3	103.8	100.4

		**Selenium Se**	**Strontium Sr**	**Molybdenum Mo**	**Cadmium Cd**	**Lead Pb**	**Mercury Hg**	**Mercury (wet weight)**	**Ratio Hg/Se**
**LoQ**	**Value**	0.085	0.002	0.004	0.002	0.002	0.087	0.014	-
	**No. <LoQ**	0	0	0	15	0	0	0	-
**Statistics**	**Minimum**	1.84	0.198	0.048	-	0.011	0.075	0.014	0.033
	**Mean**	2.60	0.960	0.079	-	0.085	0.368	0.068	0.133
	**Median**	2.60	0.690	0.079	-	0.044	0.336	0.061	0.145
	**Maximum**	3.84	4.326	0.125	-	0.219	1.240	0.182	0.323
**Recovery rate**	**DORM-3**	-	-	-	116.8	98.8	-	-	-
	**DOLT-5**	120.6	108.4	103.9	100.1	93.9	109.2	109.2	-

Reference

Helsel, D.R., 2012. Statistics for Censored Environmental Data Using Minitab® and R, 2nd ed, Statistics in practice. Wiley.

Ratcliffe, D.A., 1970. Changes Attributable to Pesticides in Egg Breakage Frequency and Eggshell Thickness in Some British Birds. J. Appl. Ecol. 7, 67–115. https://doi.org/10.2307/2401613
